# EHMTI-0059. Reduced functional connectivity between salience and visual networks in migraine with aura

**DOI:** 10.1186/1129-2377-15-S1-E41

**Published:** 2014-09-18

**Authors:** SJ Wang, D Niddam, KL Lai, JL Fuh, CYN Chuang, CYN Chuang, WT Chen

**Affiliations:** 1 Department of Neurology Facuty of Medicine, National Yang-Ming University, Taipei, Taiwan; 2 Center of Brain Research, National Yang-Ming University, Taipei, Taiwan; 3 Department of Neurology, Gan-Dau Hospital Taipei, Taipei, Taiwan; 4 Department of Neurology, Veterans General Hospital Taipei, Taipei, Taiwan

## Introduction

Migraine with visual aura (MA) is associated with distinct visual disturbances preceding migraine attacks, but shares other visual deficits in-between attacks with migraine without aura (MO). The dorsal extrastriate cortex in the occipital lobe (V3A) and middle temporal cortex were reported to be relevant neural substrate for MA and MO based on structural MRI studies.

## Aims

To determine if abnormalities specific to inter-ictal MA exist in functional brain connectivity of intrinsic cognitive networks. In particular, these networks are involved in top-down modulation of the visual system.

## Methods

Using resting-state functional magnetic resonance imaging, whole-brain functional connectivity maps were derived from seeds placed within the default mode (DMN), salience (SAL), and dorsal attention networks (DAN). Twenty-six inter-ictal MA patients were compared with 26 matched MO patients and 26 controls. Headache parameters were used for regression analyses.

## Results

The major findings were: (1) Connectivity between the SAL and occipital visual areas was reduced in MA but not MO and was negatively correlated with headache severity. (2) The V3A region exhibited decreased connectivity with the SAL and increased connectivity with the posterior DMN in MA. (3) Connectivity between the DAN and visual regions in the inferior and middle temporal cortices were increased in both MA and MO.

## Conclusions

The unique pattern of connectivity changes found in inter-ictal MA patients involved area V3A which has been implicated in aura generation. Hyperconnectivity to temporal visual regions may reflect inter-ictal deficits in visual perceptions in both MA and MO.

No conflict of interest.

